# Effects of Adjunctive Exercise Therapy in Patients with Bipolar Depression: A Pilot Multicenter Cluster-Randomized Controlled Trial

**DOI:** 10.3390/metabo16090679

**Published:** 2026-09-15

**Authors:** Fumito Hamada, Hikaru Hori, Hiroki Kumagai, Muneaki Ogata, Hiroyuki Yokoyama, Yuko Tomiyama, Hiroko Sugawara, Ryo Asada, Akito Hatanaka, Masato Masuda, Yuta Okamoto, Ryusei Hatae, Hitoshi Iida, Yoshimi Yamaguchi, Tetsuya Yoshida, Rika Yano, Leo Gotoh

**Affiliations:** 1 Department of Psychiatry, Faculty of Medicine, Fukuoka University, 7-45-1 Nanakuma, Jonan-ku, Fukuoka 814-0180, Japan; hamada2310@adm.fukuoka-u.ac.jp (F.H.); kumagaihiroki@adm.fukuoka-u.ac.jp (H.K.); mu1218ne@adm.fukuoka-u.ac.jp (M.O.); cstyokoyama@fukuoka-u.ac.jp (H.Y.); tomiyama@adm.fukuoka-u.ac.jp (Y.T.); h.sugawara.se@adm.fukuoka-u.ac.jp (H.S.); r.asada.ul@adm.fukuoka-u.ac.jp (R.A.); akito@adm.fukuoka-u.ac.jp (A.H.); masudapsy@adm.fukuoka-u.ac.jp (M.M.); y.okamoto.ry@adm.fukuoka-u.ac.jp (Y.O.); r.hatae.bn@adm.fukuoka-u.ac.jp (R.H.); iidah@adm.fukuoka-u.ac.jp (H.I.); otyamaguchi@adm.fukuoka-u.ac.jp (Y.Y.); y-tetsuya@fukuoka-u.ac.jp (T.Y.); r-yano@cis.fukuoka-u.ac (R.Y.); 2 Shiranui Hospital, 1800 Tegama, Omuta 836-0004, Japan; gleo@fukuoka-u.ac.jp

**Keywords:** bipolar disorder, bipolar depression, exercise therapy, psychosocial functioning, cognitive function, inflammatory cytokines, brain-derived neurotrophic factor, vascular endothelial growth factor-A, biomarkers

## Abstract

**Highlights:**

**What are the main findings?**

- This pilot RCT did not demonstrate significant superiority of adjunctive exercise over active stretching in bipolar depression.
- Exploratory biomarker–outcome correlations were observed, but none remained statistically significant after FDR correction; these findings should be considered hypothesis-generating.

**What are the implications of the main findings?**

- These preliminary findings may inform the design and hypotheses of future adequately powered trials of exercise interventions in bipolar depression.

**Abstract:**

**Background/Objectives**: Bipolar depression impairs psychosocial functioning, cognition, and quality of life. Evidence regarding adjunctive exercise remains limited. This pilot study evaluated the clinical and biological effects of adjunctive exercise in patients with bipolar depression. **Methods**: In this multicenter cluster-randomized controlled trial, 26 hospitalized patients were assigned by facility to treatment as usual plus supervised exercise (TAU + Ex) or supervised stretching (TAU + SC) for 6 weeks. Twenty-four completed the study (TAU + Ex, *n* = 14; TAU + SC, *n* = 10). Clinical outcomes and plasma tumor necrosis factor-alpha (TNF-α), interleukin (IL)-6, IL-1β, IL-2, brain-derived neurotrophic factor (BDNF), and vascular endothelial growth factor-A (VEGF-A) were assessed at baseline and week 6. The trial was registered with the University Hospital Medical Information Network Clinical Trials Registry (UMIN-CTR; UMIN000045877; registered on 1 December 2021). **Results**: FAST and QIDS-SR scores improved significantly in both the exercise and stretching groups after the 6-week intervention, but no significant group × time interactions were observed. No significant group, time, or group × time effects were observed for any plasma biomarker. Before correction for multiple comparisons, nominal correlations were observed between changes in IL-1β and FAST, BDNF and FAST, and VEGF-A and Spotter performance. None remained statistically significant after Benjamini–Hochberg FDR correction. **Conclusions**: Adjunctive exercise therapy was not superior to active stretching. The exploratory biomarker–outcome correlations did not remain significant after correction for multiple comparisons and should be considered hypothesis-generating. Larger, adequately powered randomized controlled trials are warranted.

## 1. Introduction

Bipolar disorder (BD) is a chronic psychiatric illness characterized by recurrent episodes of mania and depression and is associated with substantial impairments in psychosocial functioning and quality of life (QOL). Mental disorders are among the leading causes of years lived with disability worldwide, and BD contributes considerably to the global burden of disease [1]. Depressive episodes account for the majority of the illness course in BD [2,3] and are strongly associated with persistent impairments in psychosocial functioning, occupational performance, cognition, and QOL [4,5,6,7]. Consequently, the treatment goals for BD have shifted beyond symptomatic remission toward multidimensional recovery, encompassing psychosocial functioning, cognitive function, personal recovery, and overall well-being [4,5,6,7,8,9,10].

Current international guidelines recommend pharmacotherapy and psychosocial interventions as the foundation of treatment for bipolar depression [11,12,13,14,15]. However, depressive symptoms frequently recur despite appropriate pharmacological treatment, and functional recovery often lags behind symptomatic improvement [2,3,4,5]. Furthermore, long-term pharmacotherapy requires careful monitoring because of adverse effects and tolerability issues, while antidepressant use remains controversial because of the potential risk of mood switching in susceptible individuals [16]. Although structured psychosocial interventions have demonstrated clinical benefits, their implementation in routine practice is often limited by the need for specialized training and healthcare resources. Therefore, the development of safe, accessible adjunctive interventions capable of improving both symptoms and functional recovery remains an important clinical priority.

Growing evidence suggests that BD should be considered not only a disorder of the central nervous system but also a systemic disorder characterized by chronic low-grade inflammation and impaired neuroplasticity [17,18,19,20]. These biological abnormalities appear to interact through complex neuroimmune and metabolic pathways and may contribute to illness progression and persistent functional impairment.

Among the biological mechanisms implicated in BD, inflammatory cytokines and neurotrophic factors have attracted particular attention. Elevated circulating levels of inflammatory cytokines, including interleukin (IL)-6 and tumor necrosis factor (TNF)-α, have repeatedly been reported during depressive episodes, suggesting that immune dysregulation contributes to mood symptoms and functional impairment [17,21]. Brain-derived neurotrophic factor (BDNF) and vascular endothelial growth factor-A (VEGF-A), which regulate neuroplasticity, neurogenesis, angiogenesis, and neurovascular coupling, have also been associated with cognitive performance and functional outcomes in BD [18,19]. Recent systematic review evidence further supports the relevance of inflammatory cytokines and neurotrophic factors as peripheral biomarkers in BD [22]. A preliminary cross-sectional study from our research group found an association between IL-1β and QOL in patients with BD [21]. Based on this evidence, TNF-α, IL-6, IL-1β, IL-2, BDNF, and VEGF-A were prespecified in the CERF-BD protocol as biomarkers representing inflammatory and neuroplasticity-related pathways [23]. However, specific associations between individual biomarkers and clinical domains were not prespecified; these analyses were exploratory. Biological findings in BD remain heterogeneous, and the relationships between changes in peripheral biomarkers and multidimensional clinical recovery have yet to be fully elucidated.

Exercise therapy has attracted increasing attention as a promising adjunctive intervention because it may modulate inflammatory pathways and neuroplasticity. Meta-analyses in major depressive disorder have demonstrated beneficial effects of exercise on depressive symptoms [24]. Proposed biological mechanisms include modulation of inflammatory pathways [17,25,26] and enhancement of neuroplasticity [18]. In BD, however, evidence remains limited and findings have been inconsistent [27,28,29,30]. Interpretation is complicated by small samples, heterogeneous exercise protocols and clinical populations, and limited use of active control conditions. Although the Royal Australian and New Zealand College of Psychiatrists (RANZCP) and the National Institute for Health and Care Excellence (NICE) guidelines recognize exercise as a potentially beneficial adjunctive intervention, the Canadian Network for Mood and Anxiety Treatments (CANMAT), the World Federation of Societies of Biological Psychiatry (WFSBP), and the American Psychiatric Association (APA) conclude that current evidence remains insufficient for a strong recommendation because randomized controlled trials are scarce [11,12,13,14,15]. To address this evidence gap, our group previously published the study protocol for the Clinical Exercise Research in Fukuoka University for Bipolar Disorder (CERF-BD), a multicenter cluster-randomized controlled trial evaluating adjunctive exercise therapy for bipolar depression [23]. Recent studies, including metabolomic investigations, have further highlighted the potential value of biological markers for understanding disease mechanisms and treatment responses in BD [31]. Therefore, the present exploratory pilot study investigated the effects of a 6-week adjunctive exercise intervention on multidimensional clinical outcomes and peripheral biomarkers in hospitalized patients with bipolar depression and explored associations between changes in these biomarkers and clinical outcomes.

## 2. Materials and Methods

### 2.1. Study Design

This exploratory pilot study was conducted using data from an ongoing multicenter, cluster-randomized controlled trial investigating adjunctive exercise therapy for patients with bipolar depression. Cluster randomization at the facility level was employed to minimize intervention contamination within each participating institution. Ten psychiatric hospitals were randomly allocated to either a treatment-as-usual plus exercise therapy group (TAU + Ex) or a treatment-as-usual plus stretching control group (TAU + SC), and participants received the intervention assigned to their institution for 6 weeks. The study was conducted in accordance with the previously published study protocol for the Clinical Exercise Research in Fukuoka University for Bipolar Disorder (CERF-BD) trial [23]. Participant recruitment began in 2023 and is ongoing. At the time of the present analysis, 24 participants had completed the intervention and post-intervention assessments. The present report describes the findings of an exploratory pilot analysis of the ongoing trial. The study was approved by the Ethics Review Committee of the Faculty of Medicine, Fukuoka University (approval number: U22-01-005; approved on 24 January 2022) and was conducted in accordance with the Declaration of Helsinki. Written informed consent was obtained from all participants before enrollment. The study was registered with the University Hospital Medical Information Network Clinical Trials Registry (UMIN-CTR; UMIN000045877; registered on 1 December 2021).

### 2.2. Participants

**Figure 1:**
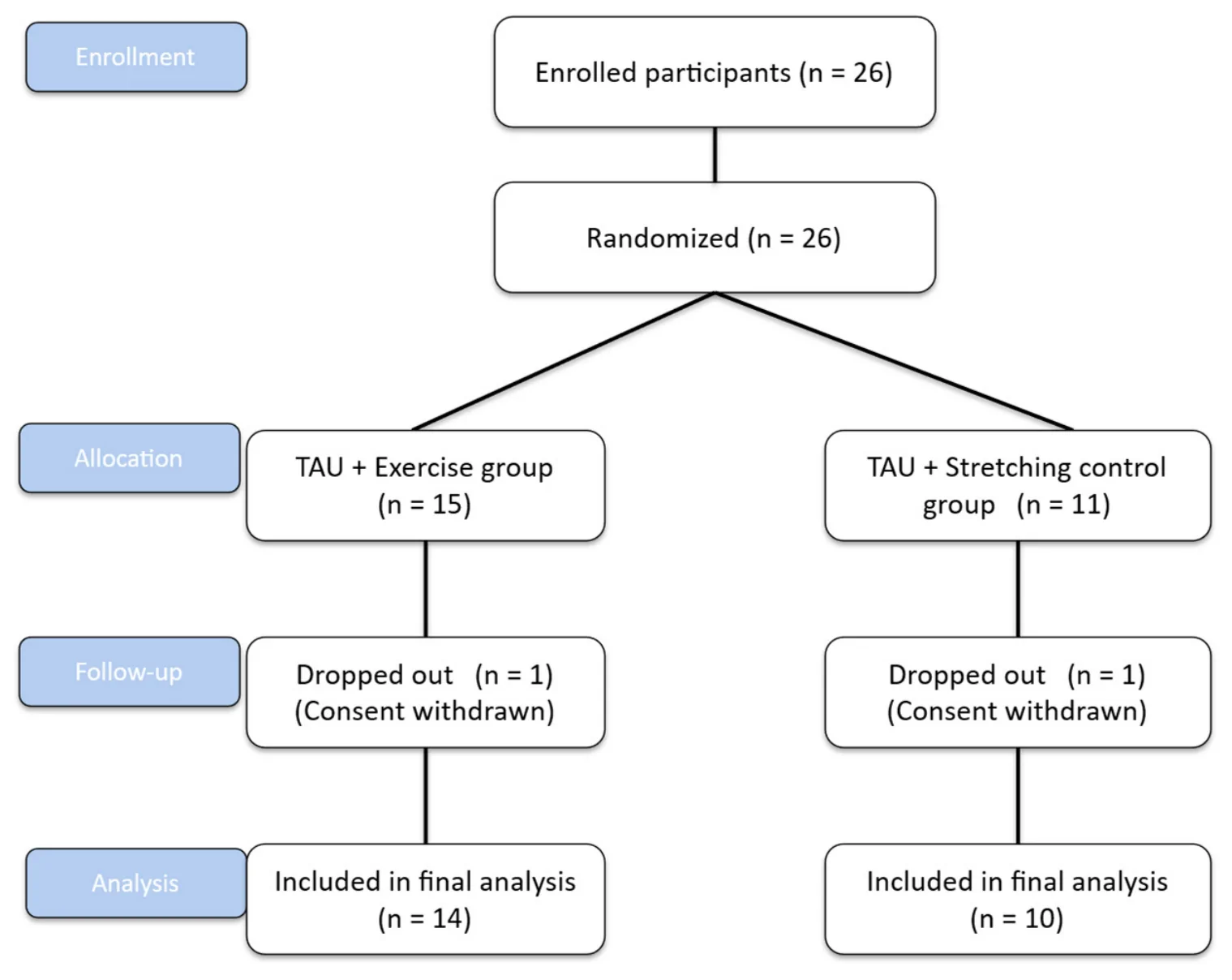
Participant flow diagram. Participant flow through enrollment, allocation, follow-up, and analysis. TAU, treatment as usual.

Participants were hospitalized patients diagnosed with BD according to the Diagnostic and Statistical Manual of Mental Disorders, Fifth Edition (DSM-5) who were experiencing a current depressive episode. Patients considered potentially eligible by their treating psychiatrists received a detailed explanation of the study and provided written informed consent before formal eligibility assessment. At the time of the present analysis, 26 participants underwent formal eligibility assessment, all of whom fulfilled the eligibility criteria and were enrolled. During the intervention period, two participants discontinued the study, resulting in 24 participants completing the intervention and post-intervention assessments (TAU + Ex, *n* = 14; TAU + SC, *n* = 10). Participant flow is presented in Figure 1.

#### 2.2.1. Inclusion Criteria

Participants were required to meet all of the following criteria:

Diagnosis of bipolar disorder according to DSM-5 criteria;
Current depressive episode;
Quick Inventory of Depressive Symptomatology–Self-Report (QIDS-SR) score between 6 and 15;
Age between 20 and 65 years;
Provision of written informed consent.

#### 2.2.2. Exclusion Criteria

Participants were excluded if they met any of the following criteria:

Intellectual disability according to DSM-5 criteria;
History of traumatic brain injury accompanied by loss of consciousness;
Pregnancy, breastfeeding, or intention to become pregnant during the study period;
Severe depressive symptoms (QIDS-SR ≥16);
Psychiatric or physical conditions judged by the treating psychiatrist to preclude safe participation in the intervention;
Any other condition judged by the principal investigator to make participation inappropriate.

### 2.3. Randomization and Blinding

Participating institutions were randomly allocated to either the TAU + Ex group or the TAU + SC group before participant recruitment, as described in the published study protocol [23]. Because of the nature of the intervention, the intervention staff and treating psychiatrists were aware of the intervention assigned to each participating institution. Participants were informed that they would be randomly assigned to one of two structured therapeutic activity programs but were not informed which program was considered the experimental intervention. The primary outcome, psychosocial functioning assessed using the Functioning Assessment Short Test (FAST), was evaluated by psychiatrists who were independent of the intervention team and blinded to treatment allocation throughout the study.

### 2.4. Interventions

#### 2.4.1. Exercise Therapy Group (TAU + Ex)

Participants allocated to the TAU + Ex group received a structured exercise program in addition to TAU for 6 weeks. Exercise sessions were conducted three times per week, with each session lasting approximately 40 min under the supervision of trained medical staff. Each session consisted of warm-up exercises, stretching of the major muscle groups (including the thighs, calves, gluteal muscles, shoulders, and back), and aerobic exercise such as walking, calisthenics, and ball games. Exercise intensity was prescribed at a light-to-moderate level, corresponding to 64–76% of the age-predicted maximum heart rate (220 − age) [32]. Perceived exertion was monitored using the Borg Rating of Perceived Exertion (RPE) Scale [33], and exercise intensity was adjusted to maintain an RPE between 11 and 13. Medical staff modified exercise intensity when necessary according to each participant’s physical condition and psychiatric status to ensure safety and adherence throughout the intervention.

#### 2.4.2. Stretching Control Group (TAU + SC)

Participants allocated to the TAU + SC group received a structured stretching program in addition to TAU for 6 weeks. Sessions were conducted three times per week for approximately 40 min under staff supervision, matching the frequency, duration, and staff contact of the exercise intervention. The program consisted of low-intensity stretching targeting the major muscle groups, including the thighs, calves, gluteal muscles, shoulders, and back. Exercise intensity was maintained below 50% of the age-predicted maximum heart rate (220 − age), with a target Borg RPE of 9–11 [33]. The stretching program was designed as an active control intervention to control for non-specific therapeutic effects, including regular staff interaction, behavioral activation, structured daily activity, and treatment expectancy, while minimizing the physiological effects of aerobic exercise.

#### 2.4.3. TAU

Participants in both groups continued to receive standard inpatient treatment for bipolar depression throughout the study, including pharmacotherapy and supportive psychotherapy in accordance with current clinical practice guidelines [11,12,13,14,15]. To minimize potential confounding, changes in psychotropic medications were not permitted during the intervention period. Participants requiring changes in mood stabilizers, antipsychotics, or antidepressants because of worsening psychiatric symptoms were withdrawn from the intervention. Additional structured psychotherapeutic interventions that could influence the study outcomes were not permitted during the intervention period.

### 2.5. Outcome Measures

Outcome assessments were performed at baseline (week 0) and immediately after completion of the 6-week intervention (week 6).

#### 2.5.1. Primary Outcome

##### Psychosocial Functioning

The primary outcome was psychosocial functioning, assessed using the Functioning Assessment Short Test (FAST) [34]. The FAST is a clinician-rated instrument comprising 24 items across six domains: autonomy, occupational functioning, cognitive functioning, financial issues, interpersonal relationships, and leisure time. Higher scores indicate greater functional impairment. FAST assessments were performed by psychiatrists who were independent of the intervention team and blinded to treatment allocation.

#### 2.5.2. Secondary Outcomes

##### Depressive Symptoms

Depressive symptoms were assessed using the Quick Inventory of Depressive Symptomatology–Self-Report (QIDS-SR) [35]. Total scores range from 0 to 27, with higher scores indicating greater depressive symptom severity.

##### QOL

QOL was assessed using the Japanese version of the World Health Organization Quality of Life Assessment (WHOQOL-26) [36,37]. The WHOQOL-26 is the Japanese version of the WHOQOL-BREF and evaluates four domains: physical health, psychological health, social relationships, and environment. Higher scores indicate better QOL.

##### Personal Recovery

Personal recovery was assessed using the Japanese version of the Questionnaire about the Process of Recovery (QPR-J) [38]. The QPR-J is a 22-item self-administered questionnaire evaluating subjective aspects of recovery, including hope, confidence, self-determination, and meaning in life. In accordance with the published correction [38], each item was scored from 0 to 4, yielding a total score from 0 to 88. Higher scores indicate better personal recovery.

##### Cognitive Function

Cognitive function was assessed using the THINC-it cognitive assessment battery [39], which includes four objective cognitive tasks and one self-report questionnaire.

1. Objective cognitive function

Spotter

Spotter assesses attention and reaction time. Mean reaction time (ms) was used for analysis, with shorter reaction times indicating better attention and processing speed.

Symbol Check

Symbol Check assesses working memory and attention using a one-back paradigm. The total number of correct responses was analyzed, with higher scores indicating better performance.

Codebreaker

Codebreaker is based on the Digit Symbol Substitution Test and evaluates processing speed, attention, visual scanning, motor speed, and working memory. Higher scores indicate better cognitive performance.

Trails

Trails is based on the Trail Making Test Part B and evaluates executive functioning. Completion time (seconds) was analyzed, with shorter times indicating better executive function.

2. Subjective Cognitive Function

Subjective cognitive function was assessed using the Perceived Deficits Questionnaire-5-Depression (PDQ-5-D). The PDQ-5-D is a self-administered questionnaire evaluating perceived cognitive dysfunction. Lower scores indicate fewer subjective cognitive complaints and better perceived cognitive functioning.

### 2.6. Blood Sampling and Biomarker Measurements

Venous blood (7 mL) was collected at baseline and week 6 by nursing personnel between 06:00 and 09:00 after an overnight fast, using Venoject^®^ II vacuum blood collection tubes (Terumo Corp., Tokyo, Japan) containing ethylenediaminetetraacetic acid disodium salt (EDTA-2Na). At week 6, the interval between the final exercise or stretching session and blood collection was not standardized. Plasma was separated by centrifugation at 2500× *g* for 20 min at room temperature within 12 h of collection and stored at −80 °C until analysis. Plasma concentrations of TNF-α, IL-6, IL-1β, IL-2, BDNF, and VEGF-A were quantified by Eurofins GeneticLab Co., Ltd. (Sapporo, Japan) in singlicate using Luminex Discovery Assay Human Premixed Multi-Analyte Kits (R&D Systems, Minneapolis, MN, USA). TNF-α, IL-6, IL-1β, IL-2, and BDNF were measured using catalog number LXSAHM-05, and VEGF-A using LXSAHM-01. Samples were kept frozen during transport. The nominal standard-curve ranges were 8.23–2000 pg/mL for TNF-α, 3.25–790 pg/mL for IL-6, 17.7–4300 pg/mL for IL-1β, 30.9–7500 pg/mL for IL-2, 20.6–5000 pg/mL for BDNF, and 11.5–2800 pg/mL for VEGF-A. Values below the measurable range were assigned 0 pg/mL for analysis. Laboratory personnel were blinded to treatment allocation.

### 2.7. Statistical Analysis

Statistical analyses were performed using JMP Student Edition, version 19 (SAS Institute Inc., Cary, NC, USA). Statistical significance was defined as a two-sided *p*-value < 0.05. Continuous variables are presented as mean ± standard deviation and categorical variables as number and percentage. Generalized linear mixed models (GLMMs) were used for continuous outcomes. For baseline comparisons, group was included as a fixed effect, study site as a random effect, and baseline mood stabilizer use as a covariate; categorical variables were compared using Fisher’s exact test. Within-group changes were evaluated using GLMMs with time as a fixed effect and participant ID and study site as random effects, adjusted for baseline mood stabilizer use. Longitudinal analyses included group, time, and group × time as fixed effects and participant ID and study site as random effects, adjusted for baseline mood stabilizer use. Mood stabilizer use remained unchanged during the intervention and was treated as a time-invariant covariate. Analyses requiring paired measurements used complete cases; missing values were not imputed. Exploratory associations between week-6 minus baseline changes in biomarkers and clinical outcomes were evaluated using Spearman’s rank correlation coefficients (ρ). Unadjusted *p*-values and Benjamini–Hochberg FDR-adjusted q-values across all 54 correlations are reported. These correlation analyses were considered exploratory and hypothesis-generating.

### 2.8. Sample Size

Because participant recruitment is ongoing, the present exploratory pilot analysis included all participants who had completed the intervention and post-intervention assessments at the time of data extraction. Consequently, no formal sample size calculation was performed specifically for this exploratory analysis.

## 3. Results

### 3.1. Participant Flow and Baseline Characteristics

A total of 26 participants were enrolled and allocated to the TAU + Ex group (*n* = 15) or the TAU + SC group (*n* = 11). One participant in each group withdrew consent before the week-6 assessment, leaving 24 participants for the final analysis (TAU + Ex, *n* = 14; TAU + SC, *n* = 10) (Figure 1). All analyzed participants attended at least 80% of scheduled sessions, and no serious intervention-related adverse events occurred. Baseline characteristics are summarized in Table 1. No statistically significant between-group differences were observed. Because baseline mood stabilizer use was numerically imbalanced, it was included as a covariate in the GLMMs.

### 3.2. Primary Outcome

Psychosocial Functioning

FAST scores improved from 28.9 ± 7.8 to 24.6 ± 10.4 in the TAU + Ex group (*p* < 0.01) and from 34.2 ± 11.4 to 28.6 ± 8.6 in the TAU + SC group (*p* < 0.01) (Table 2). The longitudinal GLMM showed a significant time effect (F(1, 21) = 30.42, *p* < 0.001), but no significant group effect (F(1, 4.70) = 0.86, *p* = 0.40) or group × time interaction (F(1, 21) = 1.03, *p* = 0.32) (Table 3).

### 3.3. Secondary Outcomes

Depressive Symptoms

QIDS-SR scores improved from 11.3 ± 2.9 to 7.9 ± 4.9 in the TAU + Ex group (*p* < 0.01) and from 10.9 ± 3.4 to 7.0 ± 3.6 in the TAU + SC group (*p* < 0.01) (Table 2). The longitudinal GLMM showed a significant time effect (F(1, 43) = 19.44, *p* < 0.001), but no significant group effect (F(1, 43) = 0.49, *p* = 0.49) or group × time interaction (F(1, 43) = 0.08, *p* = 0.78) (Table 3). Detailed results for other secondary clinical and cognitive outcomes are provided in Supplementary Tables S1 and S2.

### 3.4. Plasma Biomarkers

No significant within-group changes were observed in TNF-α, IL-6, IL-1β, IL-2, BDNF, or VEGF-A in either group (Table 2). The longitudinal GLMMs showed no significant group, time, or group × time effects for any plasma biomarker (Table 3).

### 3.5. Exploratory Correlation Analyses

Before correction for multiple comparisons, changes in FAST scores were nominally correlated with changes in IL-1β (ρ = −0.62, *p* < 0.01) and BDNF (ρ = −0.45, *p* = 0.03), and changes in Spotter performance were nominally correlated with changes in VEGF-A (ρ = −0.45, *p* = 0.03) (Table 4). None of the 54 correlations remained statistically significant after Benjamini–Hochberg FDR correction (all q > 0.05).

## 4. Discussion

This exploratory pilot study evaluated the effects of a 6-week adjunctive exercise intervention on multidimensional clinical outcomes and peripheral biomarkers in hospitalized patients with BD. FAST and QIDS-SR scores improved significantly in both the exercise and stretching groups after the 6-week intervention, but no significant group × time interactions were observed. Thus, adjunctive exercise did not demonstrate significant superiority over active stretching for these outcomes. These improvements may reflect comprehensive inpatient treatment and participation in structured therapeutic activities in both groups. The active stretching control also accounted for staff interaction, behavioral activation, and treatment expectancy. The 6-week intervention may have been too short to detect differential effects on functioning, which can recover more slowly than depressive symptoms [5,40]. Objective cognitive performance also remained largely unchanged, consistent with reports that cognitive impairment can persist beyond symptomatic improvement [41,42]. No significant group, time, or group × time effects were observed for inflammatory cytokines, BDNF, or VEGF-A. The short intervention, small sample, and concomitant pharmacotherapy may have limited the ability to detect biomarker changes. Mood stabilizers such as lithium and valproate may affect neuroplasticity and inflammatory pathways [19]; baseline mood stabilizer use was therefore included as a covariate. Several nominal biomarker–outcome correlations were observed, but none remained significant after FDR correction. These findings do not establish directionality, causality, or a biological mechanism of clinical improvement and should be considered hypothesis-generating.

The present findings should be interpreted in light of several limitations. First, the small sample limited statistical power. Randomization was performed at the facility level, and cluster sizes were small and unbalanced. Although study site was included as a random effect in the GLMMs, cluster-level variance may have been estimated imprecisely. Second, complete blinding was not feasible, although FAST was assessed by independent psychiatrists blinded to treatment allocation. Third, the intervention period was limited to 6 weeks. Fourth, pharmacotherapy was not standardized, although medication regimens remained unchanged and baseline mood stabilizer use was included as a covariate. Fifth, the interval between the final intervention session and week-6 blood collection was not standardized. Finally, numerous exploratory correlations were examined. FDR correction was applied, and none remained significant. The findings should therefore be regarded as preliminary and hypothesis-generating.

Despite these limitations, this study used a multicenter cluster-randomized design, a blinded assessment of the primary outcome, and an active stretching control. It also evaluated multidimensional clinical outcomes together with inflammatory and neuroplasticity-related biomarkers. This approach may inform future adequately powered studies of exercise interventions and potential biological correlates in BD.

## 5. Conclusions

In conclusion, this exploratory pilot study did not demonstrate significant superiority of adjunctive exercise therapy over active stretching in patients with bipolar depression. FAST and QIDS-SR scores improved over time in both groups, but no significant group × time interactions were observed. Exploratory biomarker–outcome correlations did not remain statistically significant after FDR correction and should be considered hypothesis-generating. Larger, adequately powered multicenter randomized controlled trials with longer follow-up and prespecified hypotheses are needed.

## Figures and Tables

**Table 1:** Baseline characteristics of participants.

Characteristic	TAU + Ex	TAU + SC	*p*-Value
Demographic and clinical variables			
Age (years)	41.9 ± 12.6	44.5 ± 14.0	0.86
Sex (M/F)	9/5	6/4	1.00
Number of hospitalizations	3.6 ± 2.8	4.3 ± 3.4	0.59
Education (years)	13.9 ± 2.9	12.5 ± 2.7	0.64
Age at onset (years)	30.1 ± 12.8	28.2 ± 8.8	0.73
Duration of illness (years)	11.7 ± 8.2	16.1 ± 10.8	0.29
BD I/BD II (*n*)	5/9	5/5	0.68
BMI (kg/m^2^)	27.0 ± 5.5	27.7 ± 4.9	0.78
Medication, *n* (%)			
Mood stabilizers	13 (92.9)	6 (60.0)	0.12
Antipsychotics	11 (78.6)	6 (60.0)	0.39
Antidepressants	1 (7.1)	3 (30.0)	0.27
Clinical outcomes			
FAST total score	28.9 ± 7.8	34.2 ± 11.4 (*n* = 9)	0.31
QIDS-SR total score	11.3 ± 2.9	10.9 ± 3.4	0.43
WHOQOL-26	72.8 ± 13.0	76.2 ± 14.2	0.93
QPR-J total score	43.1 ± 12.2	46.0 ± 13.0	0.63
Cognitive function (THINC-it)			
Spotter (ms)	677.2 ± 371.2	799.8 ± 250.2	0.30
Symbol Check	21.1 ± 12.8	21.6 ± 9.5	0.47
Codebreaker	45.6 ± 20.3	43.4 ± 17.8	0.40
Trails (sec)	35.8 ± 25.8	40.5 ± 19.0	0.46
PDQ-5-D	7.0 ± 3.2	6.1 ± 3.7	0.95
Biomarkers			
TNF-α (pg/mL)	2.7 ± 1.2	2.7 ± 1.5	0.67
IL-6 (pg/mL)	2.2 ± 1.4	2.9 ± 1.0 (*n* = 9)	0.11
IL-1β (pg/mL)	4.3 ± 2.7	4.4 ± 4.3	0.98
IL-2 (pg/mL)	1.1 ± 1.9	1.6 ± 3.2	0.99
BDNF (pg/mL)	5733.5 ± 5636.3	6705.7 ± 4419.2	0.75
VEGF-A (pg/mL)	160.8 ± 237.1	86.3 ± 59.2	0.08

Continuous variables are presented as mean ± standard deviation, and categorical variables as *n* (%). Between-group comparisons for continuous variables were performed using generalized linear mixed models with group as a fixed effect, study site as a random effect, and baseline mood stabilizer use as a covariate. Categorical variables were compared using Fisher’s exact test. Unless otherwise indicated, *n* = 14 for TAU + Ex and *n* = 10 for TAU + SC. FAST and IL-6 data were available for 9 participants in the TAU + SC group. Missing data were not imputed.

**Table 2:** Within-group changes from baseline to 6 weeks.

Outcome	TAU + Ex			TAU + SC		
	Baseline	6 Weeks	*p*-Value	Baseline	6 Weeks	*p*-Value
Clinical outcomes						
FAST total score	28.9 ± 7.8	24.6 ± 10.4	< 0.01	34.2 ± 11.4	28.6 ± 8.6	<0.01
QIDS-SR total score	11.3 ± 2.9	7.9 ± 4.9	< 0.01	10.9 ± 3.4	7.0 ± 3.6	<0.01
Biomarkers						
TNF-α (pg/mL)	2.7 ± 1.2	2.3 ± 1.0	0.21	2.7 ± 1.5	2.4 ± 1.0	0.46
IL-6 (pg/mL)	2.2 ± 1.4	3.0 ± 2.7	0.08	2.9 ± 1.0	3.3 ± 2.5	0.47
IL-1β (pg/mL)	4.3 ± 2.7	3.4 ± 3.5	0.41	4.4 ± 4.3	5.0 ± 2.8	0.72
IL-2 (pg/mL)	1.1 ± 1.9	1.1 ± 1.7	0.89	1.6 ± 3.2	0.2 ± 0.6	0.24
BDNF (pg/mL)	5733.5 ± 5636.3	4194.5 ± 3801.3	0.41	6705.7 ± 4419.2	5997.4 ± 5840.1	0.80
VEGF-A (pg/mL)	160.8 ± 237.1	90.7 ± 108.3	0.23	86.3 ± 59.2	85.7 ± 110.2	0.75

Data are presented as mean ± standard deviation. Within-group changes from baseline to 6 weeks were analyzed using generalized linear mixed models with time as a fixed effect, participant ID and study site as random effects, and baseline mood stabilizer use as a covariate. Mood stabilizer use remained unchanged throughout the 6-week intervention. Descriptive statistics are based on available data at each time point. Unless otherwise indicated, *n* = 14 for TAU + Ex and *n* = 10 for TAU + SC; baseline FAST and IL-6 data were available for 9 participants in the TAU + SC group. Analyses requiring paired measurements were conducted using complete cases, and missing data were not imputed. Detailed secondary clinical and cognitive outcomes are provided in Supplementary Table S1.

**Table 3:** Generalized linear mixed model results for group, time, and group × time interaction effects.

Outcome	Group F (dfN, dfD)	Group *p*-Value	Time F (dfN, dfD)	Time *p*-Value	Interaction F (dfN, dfD)	Interaction *p*-Value
FAST total score	0.86 (1, 4.70)	0.40	30.42 (1, 21)	<0.001	1.03 (1, 21)	0.32
QIDS-SR total score	0.49 (1, 43)	0.49	19.44 (1, 43)	<0.001	0.08 (1, 43)	0.78
TNF-α (pg/mL)	0.06 (1, 1.90)	0.83	2.10 (1, 22)	0.16	0.04 (1, 22)	0.84
IL-6 (pg/mL)	0.14 (1, 1.90)	0.75	2.87 (1, 21)	0.10	0.04 (1, 21)	0.85
IL-1β (pg/mL)	1.21 (1, 43)	0.28	0.03 (1, 43)	0.87	0.66 (1, 43)	0.42
IL-2 (pg/mL)	0.89 (1, 5.20)	0.39	1.16 (1, 22)	0.29	1.52 (1, 22)	0.23
BDNF (pg/mL)	0.48 (1, 1.60)	0.57	0.53 (1, 22.20)	0.48	0.07 (1, 22.20)	0.79
VEGF-A (pg/mL)	0.57 (1, 43)	0.45	0.86 (1, 43)	0.36	0.83 (1, 43)	0.37

F columns report the F statistic followed by the numerator and denominator degrees of freedom in parentheses. *p*-values are reported to two decimal places, except for values below 0.01, which are reported to three decimal places; values below 0.001 are presented as *p* < 0.001. Generalized linear mixed models included group, time, and the group × time interaction as fixed effects, participant ID and study site as random effects, and baseline mood stabilizer use as a covariate. Mood stabilizer use remained unchanged throughout the 6-week intervention. Analyses were performed using available data without imputation. Detailed results for secondary clinical and cognitive outcomes are provided in Supplementary Table S2.

**Table 4:** Exploratory correlations between changes in clinical and cognitive outcomes and changes in biomarkers.

Outcome	Statistic	TNF-α	IL-6	BDNF	IL-1β	IL-2	VEGF-A
FAST	Spearman ρ	−0.14	−0.01	−0.45	−0.62	0.00	0.18
	*p*-value	0.54	0.97	0.03	<0.01	1.00	0.42
	FDR-adjusted q-value	1.00	1.00	0.60	0.10	1.00	1.00
QIDS-SR	Spearman ρ	0.21	−0.14	0.15	−0.09	0.22	−0.03
	*p*-value	0.33	0.53	0.48	0.66	0.30	0.89
	FDR-adjusted q-value	0.89	1.00	1.00	1.00	0.85	1.00
WHOQOL-26	Spearman ρ	0.05	0.00	−0.22	−0.02	−0.30	0.03
	*p*-value	0.80	1.00	0.30	0.93	0.16	0.90
	FDR-adjusted q-value	1.00	1.00	0.88	1.00	0.85	1.00
QPR-J	Spearman ρ	−0.04	0.36	0.11	0.30	−0.06	0.06
	*p*-value	0.85	0.09	0.60	0.16	0.76	0.80
	FDR-adjusted q-value	1.00	0.80	1.00	0.79	1.00	1.00
Spotter	Spearman ρ	−0.18	0.13	−0.07	−0.31	0.29	−0.45
	*p*-value	0.42	0.56	0.76	0.16	0.19	0.03
	FDR-adjusted q-value	1.00	1.00	1.00	0.93	0.84	0.89
Symbol Check	Spearman ρ	−0.41	−0.02	−0.08	0.01	0.09	−0.08
	*p*-value	0.05	0.93	0.72	0.95	0.67	0.72
	FDR-adjusted q-value	0.73	1.00	1.00	1.00	1.00	1.00
Codebreaker	Spearman ρ	0.17	0.11	−0.28	−0.23	−0.16	0.11
	*p*-value	0.43	0.62	0.19	0.29	0.47	0.62
	FDR-adjusted q-value	1.00	1.00	0.78	0.91	1.00	1.00
Trails	Spearman ρ	−0.31	−0.04	−0.27	−0.23	−0.35	−0.06
	*p*-value	0.15	0.86	0.20	0.28	0.10	0.80
	FDR-adjusted q-value	1.00	1.00	0.79	0.96	0.76	1.00
PDQ-5-D	Spearman ρ	−0.02	−0.11	0.17	−0.01	0.24	−0.39
	*p*-value	0.93	0.64	0.43	0.95	0.27	0.06
	FDR-adjusted q-value	1.00	1.00	0.98	1.00	0.96	0.70

Spearman rank correlation coefficients (ρ) were calculated between changes from baseline to week 6 in clinical or cognitive outcomes and changes in biomarker levels. *p*-values are unadjusted. The Benjamini–Hochberg false discovery rate (FDR) procedure was applied across all 54 correlations. None of the correlations remained statistically significant after FDR correction (all q > 0.05). These analyses were exploratory and hypothesis-generating.

## Data Availability

The datasets generated and/or analyzed during the current study are available from the corresponding author upon reasonable request.

## Supplementary Materials

The following supporting information can be downloaded at https://www.mdpi.com/article/10.3390/metabo16090679/s1, Table S1: Within-group changes in secondary clinical and cognitive outcomes from baseline to week 6; Table S2: Generalized linear mixed model results for secondary clinical and cognitive outcomes.
