# Supplementary material for: Effects of Adjunctive Exercise Therapy in Patients with Bipolar Depression: A Pilot Multicenter Cluster-Randomized Controlled Trial
Source: Metabolites. 2026 Sep 15;16(9):679. doi: 10.3390/metabo16090679 (PMC13609384; doi:10.3390/metabo16090679)
Supplement: Supplementary file 1 [file metabolites-16-00679-s001.zip › metabolites-4474375-supplementary.pdf]

Supplementary Table S1. Within-group changes in secondary clinical and cognitive outcomes from baseline to 6 weeks

Outcome	TAU + Ex			TAU + SC		
	**Baseline**	**6 weeks**	**p-value**	**Baseline**	**6 weeks**	**p-value**
Secondary clinical outcomes						
WHOQOL-26 total score	72.8 ± 13.0	81.6 ± 14.2	0.04	76.2 ± 14.2	82.9 ± 12.9	0.06
QPR-J total score	43.1 ± 12.2	55.0 ± 12.6	< 0.01	46.0 ± 13.0	53.8 ± 12.7	< 0.01
Cognitive outcomes						
Spotter (ms)	677.2 ± 371.2	646.4 ± 333.5	0.74	799.8 ± 250.2	860.1 ± 444.5	0.69
Symbol Check	21.1 ± 12.8	23.9 ± 12.1	0.27	21.6 ± 9.5	20.5 ± 11.0	0.46
Codebreaker	45.6 ± 20.3	50.8 ± 18.6	0.13	43.4 ± 17.8	45.5 ± 21.6	0.59
Trails (sec)	35.8 ± 25.8	28.8 ± 17.1	0.17	40.5 ± 19.0	45.9 ± 35.5	0.49
PDQ-5-D total score	7.0 ± 3.2	3.8 ± 3.2	< 0.01	6.1 ± 3.7	4.7 ± 4.3	0.23

Data are presented as mean ± standard deviation. Within-group changes from baseline to 6 weeks were analyzed using generalized linear mixed models (GLMMs) with time as a fixed effect, participant ID and study site as random effects, and baseline mood stabilizer use as a covariate. Mood stabilizer use remained unchanged throughout the 6-week intervention. Unless otherwise indicated, n = 14 for TAU + Ex and n = 10 for TAU + SC at baseline; week-6 cognitive data were available for 13 participants in TAU + Ex. Analyses requiring paired measurements were conducted using complete cases, and missing data were not imputed.

Supplementary Table S2. Generalized linear mixed model (GLMM) results for secondary clinical and cognitive outcomes

Outcome	Group F (dfN, dfD)	Group p-value	Time F (dfN, dfD)	Time p-value	Interaction F (dfN, dfD)	Interaction p-value
WHOQOL-26 total score	0.03 (1, 1)	0.89	8.54 (1, 22)	0.008	0.15 (1, 22)	0.70
QPR-J total score	0.18 (1, 1)	0.74	17.49 (1, 22)	<0.001	0.77 (1, 22)	0.39
Spotter(ms)	2.02 (1, 2.50)	0.27	0.31 (1, 21)	0.59	0.03 (1, 21)	0.86
Symbol Check	0.04 (1, 1)	0.88	0.09 (1, 21)	0.77	0.85 (1, 21)	0.37
Codebreaker	0.92 (1, 40.90)	0.34	0.89 (1, 41)	0.35	0.03 (1, 41)	0.86
Trails(sec)	1.25 (1, 3.20)	0.34	0.07 (1, 21)	0.79	2.10 (1, 21)	0.16
PDQ-5-D total score	0.04 (1, 1)	0.87	11.50 (1, 21)	0.003	1.71 (1, 21)	0.21

F columns report the F statistic followed by the numerator and denominator degrees of freedom in parentheses. P-values are reported to two decimal places, except for values below 0.01, which are reported to three decimal places; values below 0.001 are presented as $p < 0.001$. Generalized linear mixed models (GLMMs) included group, time, and the group × time interaction as fixed effects, participant ID and study site as random effects, and baseline mood stabilizer use as a covariate. Mood stabilizer use remained unchanged throughout the 6-week intervention. Analyses were performed using available data without imputation.
